# The Influence of Initiators, Particle Size and Composition on the Electrokinetic Potential of N-(Isopropyl)acrylamide Derivatives

**DOI:** 10.3390/polym16070907

**Published:** 2024-03-26

**Authors:** Monika Gasztych, Aleksandra Malamis, Witold Musiał

**Affiliations:** Department of Physical Chemistry and Biophysics, Pharmaceutical Faculty, Wroclaw Medical University, Borowska 211, 50-556 Wroclaw, Poland; monika.gasztych@umw.edu.pl (M.G.); amalamiss@gmail.com (A.M.)

**Keywords:** NIPA, polymer, thermosensitive, zeta potential

## Abstract

The aim of this study was to characterize and compare the zeta potential of particles sensitive to external thermal stimuli. Poly N-(isopropyl) acrylamide (PNIPA) was selected as the thermosensitive polymer with a volume phase transition temperature (VPTT) between 32 and 33 °C. The hydrodynamic diameter (D_H_) of the nanoparticles was measured by dynamic light scattering. Zeta potential (ZP) measurements were performed with the same instrument used for D_H_ measurements. ZP measurements allow the prediction of the stability of colloidal systems in aqueous solutions. These measurements were combined with a pH study before and after the purification process of the particles. The ZP was measured to determine the electrostatic interactions between the particles, which can lead to particle aggregation and decrease their colloidal stability. The effect of the composition of the synthesized particles on the ZP was assessed. One of the most important factors influencing ZP is pH, especially in aqueous solutions. The initiator did not significantly affect the D_H_ of the particles, but it did significantly affect the ZP. The synthesized particles were subjected to a visible radiation absorption study in the selected temperature range to determine the VPTT.

## 1. Introduction

Polymeric nanoparticles are often studied as structures that enable the transport of an active pharmaceutical ingredient (API); they can protect the API during the degradation process or influence the metabolic pathway, but the nanoparticles can also affect the solubility of the API [1,2]. Nanoparticles are used in the manufacture of innovative model drugs for controlled drug release. This topic is of interest due to the potential beneficial influence of this type of drug carrier on the safety and efficacy of pharmacological therapy [3]. Sensitive polymer research is an emerging scientific discipline. Sensitive polymers have great potential in a wide range of applications and show sensitivity to various stimuli and small changes in environmental factors [4,5]. One of their key benefits is the ability to deliver drugs and genes precisely and in a targeted manner [6,7]. Smart polymers are used to release medical substances at appropriate times and concentrations as a result of a specific stimulus activity, including pH, temperature, light, electric or magnetic field [8]. This type of particle exhibits a non-linear response to a given stimulus, resulting in a change in its structure. The response to the stimulus may be based on polymer swelling, polymer network collapsing or macromolecular disruption [9]. Different temperatures lead to changes in the polymer configurations, resulting in changes in the amount of drug released [10].

**Figure 1:**
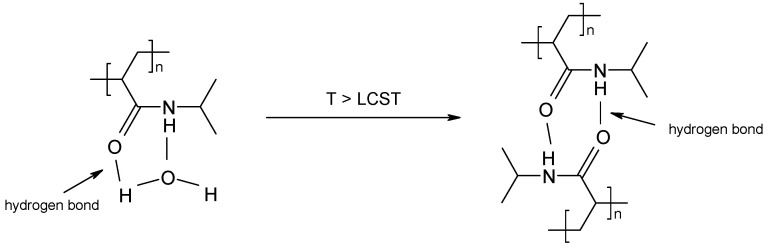
Mechanism of LCST transition of PNIPA. The dotted line represents the hydrogen bonds [16].

Our scientific team studies the synthesis and physico-chemical properties of sensitive polymers. The most interesting polymers are thermosensitive polymers, especially those with characteristic thermal properties close to physiological temperature. Therefore, we highlight the thermosensitive polymer poly N-(isopropyl) acrylamide (PNIPA) with its sharp lower critical solution temperature (LCST) of about 32–33 °C, which is close to biocompatible and has been of interest to scientists for many years. PNIPA can be used as a component in controlled release systems [11,12]. Temperature variations cause changes in the polymer configuration and modulate the release rate of the drug [13]. Below a certain temperature, PNIPA is in a specific hydrated state and is consequently soluble. Above this temperature, the polymer tends to be insoluble. Below the LCST, the polymer exists in a solvation sphere with hydrogen bonds between the hydrophilic polymer chain fragments and water molecules. After heating the system to temperatures above the LCST, the solvation sphere is disrupted and hydrophobic interactions begin to dominate, resulting in polymer precipitation (Figure 1). Crosslinked NIPA-based hydrogels swell below the critical temperature and shrink above this temperature, which is known as the volume phase transition temperature (VPTT) [14,15].

**Figure 2:**
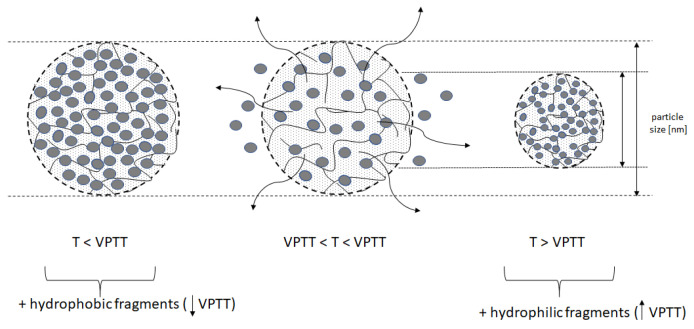
Diagram illustrating the impact of additional groups on VPTT (volume phase transition temperature) and size of the particle.

The ability to modify the VPTT is essential for the application of sensitive polymers [17]. The VPTT can be modified by controlling the substrate composition and reaction conditions, resulting in a structural modification of the polymer [18,19]. The value of VPTT depends on the content of hydrophilic and hydrophobic moieties in the particle and on their distribution [20]. It also depends on the type and concentration of the cross-linking agent used as a comonomer in the polymerization process. Incorporation of hydrophilic fragments into the polymer chain increases the VPTT, while incorporation of hydrophobic fragments reduces this temperature [21,22]. A diagram illustrating the effect on VPTT is shown in Figure 2.

The ZP is an important parameter for characterizing particles and reflects the repulsive force of the colloidal particles. The ZP depends on the chemical composition of the surface of the particle as well as the type of solvent and the ions present in the suspension. A decrease in ZP results in an increase in ionic strength [23]. pH is one of the most important factors affecting ZP, especially in aqueous solutions where H^+^ and OH^−^ ions are the main components [24]. Tae-Hwan Kim et al. investigated the effect of cationic surfactant on the ZP value [25]. Anionic surfactants influence the ZP values; sodium dodecyl sulphate (SDS) or ammonium lauryl sulphate (ALS) were used as additives in a bentonite suspension at different concentrations. The absolute ZP decreased with increasing surfactant concentration and hydrogen bonds may be formed between the particle surface and the surfactant molecules [26]. The research group characterized polysaccharide multi-nanolayers with modified physicochemical properties that influenced ZP. In this experiment, the ZP was affected by different concentrations of polysaccharides, different pH values and ionic strength. The different NaCl concentrations affected the ionic strength and consequently the ZP values. In addition, this work found that pH had a much greater effect on ZP than ionic strength [27]. The ZP is an important factor in effective endocytosis. The positively charged particles can undergo effective endocytosis by interacting with the negatively charged cell membrane [28]. 

**Figure 3:**
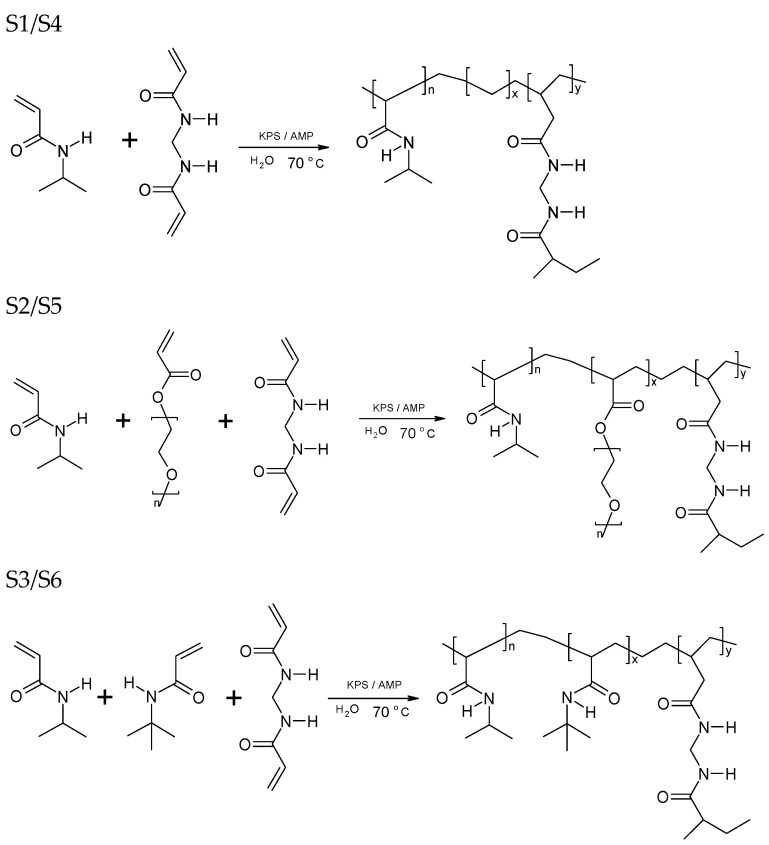
The general scheme of PNIPA derivative polymerization under the experimental conditions used in this study.

The aim of this study was to investigate the effect of the composition of the synthesized particles sensitive to external thermal stimuli on the values of ZP, VPTT and size. The particles were partly characterized in previous studies in our group [29,30,31,32]. A schematic of the synthesis of the PNIPA derivatives described in the manuscript is shown in Figure 3.

## 2. Materials and Methods

### 2.1. Materials

N-isopropyl acrylamide (NIPA, 97%, Sigma Aldrich, Sternheim, Germany), potassium persulfate (KPS, 98%, Sigma Aldrich, Sternheim, Germany), dihydrochloride 2,2′-azobis (2-methyl propionamidine) (AMP, granules, 97%, Sigma Aldrich, Sternheim, Germany), N,N-methylene bisacrylamide (MBA, 99%, Sigma Aldrich, Sternheim, Germany), poly(ethylene glycol) methyl ether acrylate (PEG-MA, 99%, average Mn 500, Sigma Aldrich, Sternheim, Germany), and N-tertbutyl acrylamide (NTB, 99%, Acros Organics, Geel, Belgium) were obtained from industrial and commercial sources and were used without further purification. The dialysis bag with a molecular mass cut-off MWCO 12,000–14,000 Da was obtained from Visking Medicell International Ltd. (London, UK). Deionised water was obtained from an ionic column and conformed to the European Pharmacopoeia monograph on purified water.

### 2.2. Methods

#### 2.2.1. Particle Synthesis

The evaluated polymers were synthesised by surfactant-free precipitation polymerisation (SFPP) without the use of an emulsifier [33]. The synthesis was carried out in a specially prepared reactor. The reactor consisted of a three-necked flask placed in a water bath. During the synthesis process, the appropriate temperature of 70 °C was maintained and monitored by a temperature sensor immersed in the reacting mixture. The reactor was combined with an Allihn reflux condenser and maintained under a nitrogen atmosphere. An appropriate volume of deionised water at 70 °C was added to the reactor. The required initiators, KPS or AMP, were dissolved in 100 mL of deionised water and added to the reactor flask. Once the temperature of the mixture had stabilised, the remaining relevant reagents were dissolved in deionised water and added to the vessel containing the reactive solution. The composition of the synthesised particles is given in Table 1 and the molar ratio of monomer to initiator is given In Table 2. Upon completion of the synthesis, a purification process of the obtained products was carried out to remove unreacted monomers, cross-linking agents and initiators by equilibrium dialysis. The purification process consisted of conductivity measurements of the acceptor liquid, which was deionised water, every 24 h until a constant conductivity value was reached. A dialysis membrane was used for this purpose. The measurements were carried out with an ELMETRON CPC-511 (Gliwice, Poland) and the conductive electrode ELMETRON EC-70t (Zabrze, Poland) was used for conductivity measurements. After the purification process, the samples were freeze-dried in a Steris Lyophilizer Lyovac GT2 for further measurements.

#### 2.2.2. Hydrodynamic Diameter Measurements

The hydrodynamic diameters (DH) of the water dispersions of the particles obtained after purification were measured by dynamic light scattering (DLS) using a Malvern Zetasizer Nano ZS ZEN 3600 (Malvern Instruments, Malvern, UK) at a wavelength of 678 nm and an angle of incidence of 90° using a polystyrene cuvette. Particle samples were diluted tenfold with deionised water. The polymer samples were evaluated using the backscattering measurement system at 173° and correlated with the parameters of the Mark–Houwink equation. Five measurements were taken for each sample and the results were analysed using Zeta Sizer Nano software version 5.03 (Malvern Instruments, Malvern, UK).

#### 2.2.3. Zeta Potential and pH Measurements

Zeta potential (ZP) measurements were performed in parallel with the DH measurements using the same instrument. For the ZP measurement, the capillary-cell-type DTS-1070 was used. Both measurements were carried out in the temperature range 18–42 °C in unbuffered aqueous solution. The pH test was carried out by the potentiometric method using a CX-741 ELMETRON multifunction computer meter (Zabrze, Poland) with an ERM-13-6 electrode (Zabrze, Poland).

#### 2.2.4. Volume Phase Transition Temperature

A UV–Vis 8453 spectrophotometer (Agilent, Santa Clara, CA, USA) connected to a Brookfield TC-101 thermostat was used to determine the volume phase transition temperature (VPTT) of the particles obtained. Samples containing 3 mL of purified polymers were prepared. Some samples were diluted tenfold with deionised water due to their intense opalescence. The cuvette containing the polymer dispersion was placed in a spectrophotometer and thermostatted. The absorbance was recorded in the wavelength range 190–1100 nm and the values obtained at 480 nm were used. The first measurement was taken at 25 °C after the temperature had stabilised. After measuring all samples at this temperature, the temperature was increased by 1 °C until 45 °C was reached.

## 3. Results

### 3.1. Hydrodynamic Diameter and Volume Phase Transition Temperature Evaluations

**Figure 4:**
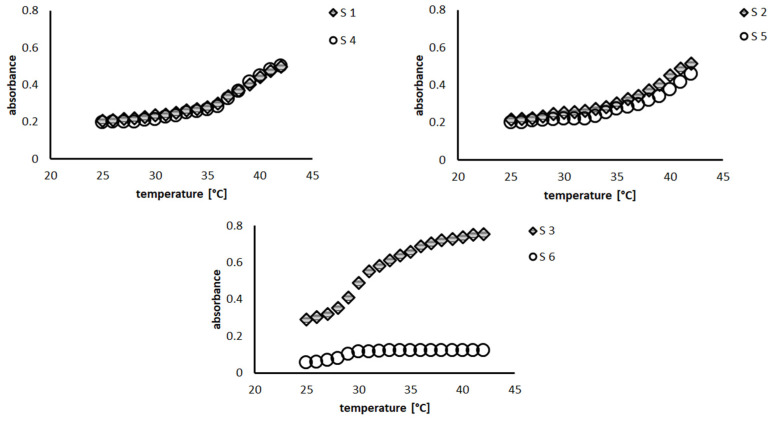
Absorbance variability in the temperature range 25–42 °C. Each chart represents two polymers with the same composition but with different initiators used during the synthesis.

The results of D_H_ at 18 and 42 °C are presented in Table 3. The D_H_ at 18 °C of the particles obtained by SFPP with the anionic initiator ranged from 525.00 nm to 859.40 nm. However, when the cationic initiator was used, the D_H_ was characterized by varied values ranging from 312.00 nm to 917.27 nm. At temperatures above the VPTT of 42 °C, a D_H_ range of 100.70 nm–413.20 nm was observed. However, the value of D_H_ was significantly lower after the system was heated. The VPTT of each particle is shown in Table 3 and in Figure 4. The lowest value of 26 °C was observed for particle S6. 

### 3.2. Zeta Potential and pH Measurements

The zeta potential at 18 °C ranged from −21.40 mV to −6.41 mV with the use of an anionic initiator. On the other hand, the cationic initiator resulted in positive values of ZP ranging from 3.70 mV to 27.40 mV. The electrokinetic potential values increased at an elevated temperature of 42 °C. The ZPs of the anionic initiator group ranged from −25.63 mV to −29.38 mV. After using a cationic, alkaline initiator, the ZPs ranged from 31.50 mV to 47.97 mV. Measurements of the pH were carried out before and after the purification process. Before the purification process (BPP), the pH values ranged from 2.24 to 5.51, while after the purification process (APP), the pH values ranged from 3.57 to 9.43. The pH changes were useful for assessing the degree of purification of the compound. The results of ZP at 18 and 42 °C and the pH test are presented in Table 4. A pH test in the 18 °C temperature was performed on all components used for particle synthesis and the results are presented in Table 5.

## 4. Discussion

**Figure 5:**
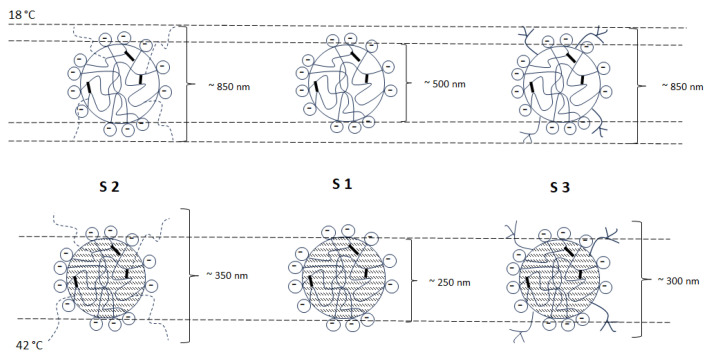
The schematic structure of synthesized particles S1–S3 with the charge results from the presence of anionic groups in the structure of the particle and the hydrodynamic diameter.

The SFPP process enables the formation of various products, including microspheres and nanospheres [33]. Through the use of a suitable polymerization technique, microspheres or nanospheres with a surface-modified core can be obtained depending on the monomer core. Through a series of syntheses, various types of small nano- and micro-sized particles were obtained. The D_H_ of the particles ranged from 100.70 nm to 917.27 nm. As a result of the precipitation polymerization, the particles exhibited a spherical shape. Particles with the anionic initiator exhibited negative electrokinetic surface potentials. Even in the absence of ionized groups, neutral surfaces can obtain surface charge by adsorbing ions from the solution. In addition, functional group impurities can affect the charge. The cationic initiator resulted in a positive charge on the surface of the nanoparticles [34,35]. The positive or negative particle surface charge is attributed to the particular initiator residues at the end of the polymer chain. Schematic structures with charge deposition on the surface and D_H_ of synthesized particles S1–S3 are shown in Figure 5.

**Figure 6:**
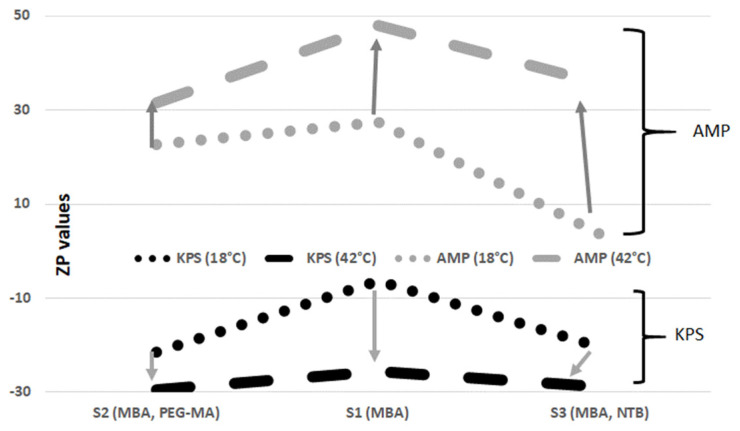
Zeta potential values as a function of temperature at 18 and 42 °C, particle composition and initiator used.

Another very important factor in the characterization of the particles was ZP. The ZP differences were evaluated in relation to the composition of the prepared particles. The ZP values at 18 and 42 °C depend on the polymer composition and are shown in Figure 6. The easily visible differences of ZP may depend on the amount of the bound initiator, as the KPS initiator as well as the AMP are permanently attached to the chain and affect the electrical charge. The positive charge on particles S4–S6 is due to the amine groups of the cationic initiator AMP. Conversely, the negative charge on the surface of particles S1–S3 comes from the terminal sulphate groups of the anionic initiator KPS [36,37]. The ZP differences depend also on the qualitative properties of the monomer and initiator, on the degree of exposure of the ionized groups in the polymeric net and on the influence of the polymer chain on the electrostatic charge system affecting the dissociation of acidic or alkaline functional groups.

**Figure 7:**
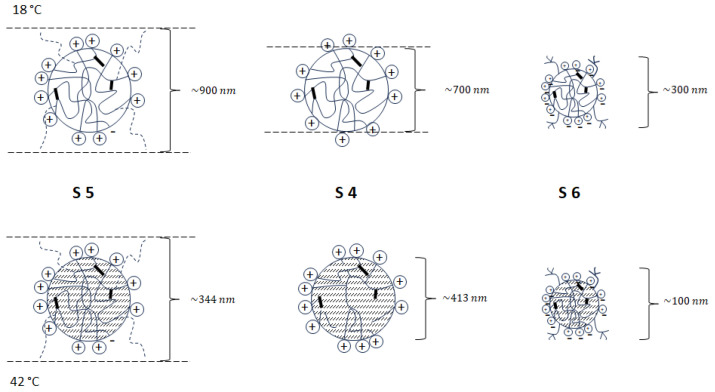
The schematic structure of synthesized particles S4–S6; with the charge results from the presence of anionic groups in the structure of the particle and the hydrodynamic diameter.

The ZP is proportional to the charge of the particle and inversely proportional to the surface area of the particle. It can be assumed that as the temperature increases, the charge does not change significantly while the surface area of the particle decreases. Therefore, the resulting ZP can increase with increasing temperature, not only because of the increased thermodynamic activity of the solvated molecules, but also because of the decreased internal friction. The decrease in internal friction results from the decrease in particle size. ZP is generated by the core of the sphere itself, with no protruding chains of additional comonomers in the molecule. The ZP charge is calculated per sphere area, with larger spheres having a lower potential and smaller spheres having a higher potential. The change in ZP is due to a change in the D_H_ core of the sphere. Schematic structures with surface charge deposition and D_H_ of synthesized particles S4–S6 are shown in Figure 7.

The ZP value indicates the potential stability of the colloidal system. Particles with an absolute ZP value higher than 30 mV are considered stable [38,39]. The speed of a molecule is influenced by several factors, including the zeta potential. Electrophoretic mobility refers to the speed at which a particle moves in an electric field. Henry’s equation can then be used to determine the zeta potential of a molecule once its electrophoretic mobility has been determined.

$$U_{E}\frac{2\varepsilon zf(K_{a})}{3\eta }$$

z—Zeta potential;
U_E_—Electrophoretic mobility;
Ɛ—Dielectric constant;
η—Viscosity.

Typically, two approximations of the function f(Ka) are used, namely 1.5 or 1.0. Electrophoretic determination of the zeta potential is mainly used in aqueous solutions with moderate electrolyte concentrations. In such cases, the function f(Ka) assumes a value of 1.5 and is referred to as the Smoluchowski approximation [39].

The characterization of particles by parameters such as Dh, ZP or VPTT is very important in the design of new drug forms. These parameters are monitored in order to assess the stability and shelf life of the product and also to obtain a drug form with the best drug-release properties. It has also been found that the DH and ZP modulations of a particle have a strong influence on the permeation-enhancing properties [40,41,42].

**Figure 8:**
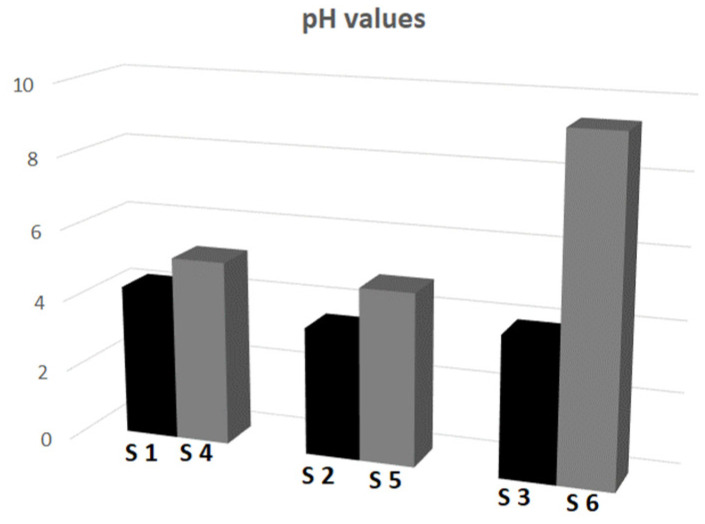
The variability of the pH values of particles S1–S6 at 18 °C, polymers synthesized with the anionic initiator (black columns, left in pair), and polymers synthesized with the cationic initiator (grey columns, right in pair).

One of the important factors affecting the ZP is the pH. An evaluation of the pH may be useful for the assessment of the degree of purification of a compound in the course of equilibrium dialysis. The pH measurements of BPP confirm that the high concentration of protons in the solution results from post-reactive impurities, i.e., unreacted monomers, comonomers or initiators. The main factors affecting pH are primarily functional groups in the particles, polarization effects and environmental resistance resulting, e.g., from increased viscosity [43,44]. In the evaluated particles, the alkaline pH results from the amide groups in NIPA. The presence of anionic groups increases the [H^+^] concentration in the system, while the labile azo groups in the cationic initiator cause an increase in the pH value [45]. The variability of the pH of S1–S6 particles is shown in Figure 8.

At temperatures below VPTT, the particles can be considered less stable due to their lower ZP values. However, at low temperatures, they form a hydrated colloidal system. At temperatures above VPTT, local dehydration occurs and the surface charge of the molecule is responsible for the stability of the colloidal system. Therefore, the ZP results measured at 42 °C are more valuable for practical purposes. It was observed that in the presence of the MBA cross-linker and AMP initiator alone, the particles produced higher ZP values than particles with MBA and other comonomers. The MBA crosslinker may restrict the movement of the polymer chains, bringing them closer together. The reason for this result is unclear, but Fuciños C. et al. attribute it to the effect of soft particle elasticity on permeability. They claim that a material with a small dielectric constant is negatively charged because there is an electric potential between two materials with different permeabilities [46]. The cores in the PNIPA-co-MBA spheres synthesized in our group are of similar size, while the additive comonomers hinder particle mobility due to increased high DH via longer, branched functionals. At higher temperatures, the particle diameter decreases and the macromolecule moves faster, resulting in higher ZP values.

Paired synthesized particles, differing only in the initiator used, are described below.

Polymer S1 was characterized by a ZP of about −25.63 mV. The D_H_ of these particles did not exceed 256.60 nm. The dimensions of the S4 microspheres were 413.20 nm, which was slightly higher than that of the S1 particles. Therefore, the initiator had little effect on the D_H_ of the resulting products, but had a significant effect on the ZP, which was 47.97 mV in the case of S4. These particles, which differed only in the initiator, showed the greatest difference in their ZPs.

By using a hydrophilic comonomer containing nine ethoxy groups in the synthesis of S2, it was possible to increase the D_H_ of the microspheres to approximately 361.24 nm. A negative charge was maintained on the surface of these particles. The use of PEG-MA in the synthesis of S5 resulted in spheres characterized by a D_H_ of 344.20 nm. The ZP remained at a value of 31.50 mV, comparable to S4. The difference in absolute ZP between S2 and S5 is practically imperceptible and it can be seen that cationic and anionic initiators have the same effect on MBA crosslinked particles as adding the hydrophilic PEG-MA comonomer. Comparing the polymer pairs S1–S4 and S2–S5, the change of initiator from anionic to cationic resulted, as expected, in an increase in the absolute ZP value as well as in an increase in the pH. This change is mainly due to the presence of the azo group from the AMP, resulting in tertiary radicals. S2 and S5 were characterized by the presence of long chains with nine ethoxy groups, which probably resulted in the relatively high D_H_ and ZP values. When the LCST temperature is exceeded, the chains may adhere to the surface of the beads, resulting in a large decrease in D_H_. The ZP values indicate the stability of the particles.

The addition of a lipophilic NTB comonomer to the main monomer in S3 resulted in microspheres with a D_H_ of 276.74 nm, similar to S1 particles. Based on the above observations, it can be concluded that the addition of PEG-MA or NTB increased the D_H_; however, the use of NTB resulted in a lower D_H_ of 100.70 nm for S6. The addition of more hydrophobic chains results in greater water displacement and significant shrinkage of the molecule. The molecule shrinks more when NTB is added, presumably due to stronger hydrophobic interactions between the hydrophilic part of the comonomer and the initiator, than when PEG-MA is added to the comonomer molecule. The lipophilicity of the comonomer has a strong influence on the ZP and D_H_ values [47,48]. The absolute ZP value of S3 was 28.87 mV and that of S6 was 35.73 mV. As in the previous case, the difference in the ZPs of the particles was limited and the effect of the lipophilic comonomer on the ZP was similar for the polymers synthesized with cationic or anionic initiators. The addition of NTB reduced the D_H_. The presence of NTB in the particles synthesized with AMP increased the pH and may be associated with an increase in the number of proton acceptor sites in the macroparticle and the formation of acidic functional groups that pass through the semipermeable membrane into the acceptor solution during the purification process.

The S3 and S6 products contained fragments of NTB on their surface. As a result of exceeding the VPTT, there was a decrease in D_H_, but not as great as in the case of S2 and S5. This can be attributed to the weaker adhesion of the NTB chains to the particle surface due to the hydrophobic properties of NTB. The high stability of particle S6 at 42 °C can be attributed to specific hydrophobic interactions between the comonomer and the crosslinker chains.

In this manuscript, two methods are used to determine the VPTT values of the collected particles. In the Zetasizer measurements, the applied wavelength of 678 nm is in the red region of the visible spectrum. In the spectrophotometer measurements, the applied wavelength of 480 nm is in the opposite region, i.e., the blue region of the visible spectrum. The difference between these two electromagnetic wavelengths did not affect the recorded VPTT value. This may be due to the proximity of the two wavelengths compared to the size and movement of the particles being assessed. The VPTT results obtained are consistent when measured by both methods.

## 5. Conclusions

The NIPA derivatives obtained via SFPP were in the nanosize range of 100.70–917.27 nm depending on the components used during polymer synthesis. The synthesized thermosensitive polymer exhibited VPTT values in the range of 26–36 °C. Particles obtained with the anionic initiator were characterized by a negative value of the ZP, which indicated the existence of a negative charge on the particle surface. The use of a cationic initiator in nearly all the samples resulted in a positive charge on the nanoparticle surface. The initiator did not influence the D_H_ of the particles; however, it significantly affected the ZP. Implementation of selected functional groups into the PNIPA molecule may enhance the stability of the particles. The use of PEG-MA in the presence of both anionic and cationic initiators resulted in relatively high ZP values, which supported the increased stability of the PNIPA particles. The addition of more hydrophobic chains resulted in greater water displacement and significant shrinkage of the molecule. The shrinkage of the molecule is greater when NTB is present.

## Figures and Tables

**Table 1:** Qualitive and quantitative compositions of the evaluated particles.

Substrates % (*w*/*w*)	Monomer	Initiator		Crosslinker	Comonomer		Solvent	Bibliography *
		Anionic	Cationic	Short Chain	Hydrophilic	Lipophilic		
Type of Polymer	NIPA	KPS	AMP	MBA	PEG-MA	NTB	H_2_O	
S1	0.5	0.05		0.05			99.40	[29]
S2	0.5	0.05		0.05	0.05		99.35	
S3	0.5	0.05		0.05		0.05	99.35	
S4	0.5		0.05	0.05			99.40	[30]
S5	0.5		0.05	0.05	0.05		99.35	
S6	0.5		0.05	0.05		0.05	99.35	

NIPA N-isopropyl acrylamide, KPS potassium persulfate, AMP dihydrochloride 2,2′-azobis (2-methyl propionamidine), MBA N,N-methylene bisacrylamide, PEG-MA poly(ethylene glycol) methyl ether acrylate, NTB N-t-butyl acrylamide; H_2_O deionized water. * Polymer compositions from previous studies that have already been published by our scientific group. In this manuscript, selected physicochemical studies of synthesized particles have been expanded.

**Table 2:** Compositions of S1–S6 microparticles and the molar ratio of monomer to initiator.

Type of Polymer	Monomer (mol)	Anionic Initiator (mol)	Cationic Initiator (mol)	NIPA to KPS/AMP Radical Molar Ratio
	NIPA	KPS	AMP	
S1–S6	4.4 × 10^−2^	1.85 × 10^−3^	1.84 × 10^−3^	1:0.1

**Table 3:** Hydrodynamic diameter (D_H_), polydispersity index (PI) and volume phase transition temperature (VPTT) values of the evaluated particles.

Type of Initiator	Type of Polymer	18 °C				42 °C				VPTT
		D_H_ [nm]	SD	PI	SD	D_H_ [nm]	SD	PI	SD	°C
anionic initiator	S1	525.00	8.61	0.21	0.02	256.60	0.20	1.31	0.01	34
	S2	838.20	10.44	0.32	0.03	361.24	2.17	0.02	0.01	35–36
	S3	859.40	17.23	0.03	0.02	276.74	1.06	0.02	0.01	29
cationic initiator	S4	724.68	4.60	0.04	0.01	413.20	3.79	0.02	0.01	35
	S5	917.27	15.81	0.35	0.01	344.20	4.18	0.04	0.03	35–37
	S6	312.00	12.48	0.49	0.01	100.70	8.24	0.35	0.02	26

**Table 4:** Zeta potential (ZP) and pH of the evaluated particles (pH at 18 °C temperature).

Type of Initiator	Type of Polymer	18 °C		42 °C		pH_BPP_	SD	pH_APP_	SD
		ZP [mV]	SD	ZP [mV]	SD				
anionic initiator	S1	−6.41	0.23	−25.63	0.14	2.58	0.08	4.22	0.07
	S2	−21.40	0.44	−29.38	0.36	2.24	0.07	3.57	0.10
	S3	−20.33	0.47	−28.87	0.90	2.38	0.03	3.94	0.12
cationic initiator	S4	27.40	0.53	47.97	0.23	5.11	0.11	5.17	0.09
	S5	22.70	0.80	31.50	0.26	4.51	0.09	4.83	0.05
	S6	3.70	0.01	35.73	0.99	5.51	0.04	9.43	0.30

**Table 5:** pH values of the substrates used in participle synthesis at 18 °C temperature.

Substrate	NIPA	KPS	AMP	MBA	PEG-MA	NTB
pH	6.23	4.23	5.86	5.15	4.25	4.14
SD	0.32	0.05	0.59	0.07	0.01	0.11

## Data Availability

Data are contained within the article.
